# A Treatise on the Diseases and Special Hygiéne of Females

**Published:** 1845-04

**Authors:** 


*A Treatise on the Diseases and Special Hygiène of Females.* By Colombat De L'Isère. Translated from the French, with Additions. By Charles D. Meigs, M. D., Professor of Midwifery and the Diseases of Women and Children in Jefferson Medical College, Philadelphia, &c., &c.; 8vo., pp. 719, with wood cut illustrations. Philadelphia, Lea & Blanchard, 1845.

"The American Publishers inherit, in an eminent degree, the peculiarities of their nation. They are an enterprising, 'go-ahead' generation. No sooner is a work of merit, or one from an author of reputation, ushered into the world, than forthwith, by the instrumentality of indifferent types, and worse paper, it is disseminated throughout the length and breadth of the New World. The most common and popular plan of proceeding, is to append notes by some writer whose name is familiar to American ears, 'so as to bring the work to a level with the state of knowledge in the United States.'"—*London and Edinburgh Monthly Journal, Feb.* 1845.

The simultaneous publication of the above works, on the same subjects and by the same house,—close, too, on the heels of the Cyclopædia of Practical Medicine, Cooper's large works, and numerous other valuable publications, several of which belong to the same department of practical medicine,—presents a strong confirmation of the foregoing remarks of the transatlantic critic. That "no sooner is a work of merit, or one from an author of reputation, ushered into the world, than forthwith" "it is disseminated throughout the length and breadth of the New World," is a compliment to the reading habits of American physicians, greater than we are accustomed to hear from that quarter, and one which we should be glad to reciprocate. As to the "indifferent types and worse paper," we must confess that we are yet somewhat at fault, but are rapidly mending even in that respect—indeed, some of the American reprints of British works, have actually exceeded the originals in these very particulars. The publications now before us, if neither costly nor splendid, are, at least, respectable in all that relates to their mechanical execution.

The practice alluded to, of appending notes "by some writer whose name is familiar to American ears," is certainly becoming a "common and popular plan," but one to which we can see no possible objection, provided the notes are by those who are conversant with the subjects on which they write, and are apposite. That *all* recent American editions of British works answer to this description, we are far from pretending. In some instances which we could point out, neither the pursuits and experience of the editors, nor the amount or character of their additions, or any other consideration than the vanity of having their names blazoned forth on the title page, would seem to afford the least pretext for such a display. However, the evil is one that speedily cures itself. Publishers are not slow in discovering the value of a name, where a work is to be pushed forward in the face of the competition that now prevails in medical book publishing.

Dr. Ashwell has been long before the public as a devoted cultivator of the subjects embraced in the present treatise, and although we discover in his writings nothing particularly striking or original, they afford evidence of judicious and careful observation. In the work before us, we have a clear, and it appears to us, a faithful exposition of the views of British practitioners on the subjects which it embraces. The diseases of pregnancy and the puerperal state, are not included. This omission, with those who are unable or disinclined to purchase separate treatises on these subjects, will constitute an objection.

The editor has found his task an easy one. So perfect, in his estimation, we presume, is the text, that he has abstained from making any material additions to it. His notes are neither numerous nor extensive.

The other work, of which we have cited the title, is altogether of a different character. Unlike Dr. Ashwell, M. Colombat has hitherto enjoyed no large share of reputation as a medical writer, particularly in reference to the subjects embraced in the present treatise; nor do we discover in it proof that any great amount of the information which it contains is drawn from his own observation. Nevertheless, it is a valuable compilation, in which the views of the ancients and of modern French writers are embodied and arranged with clearness. Like most of his countrymen, however, M. Colombat has shown but little acqaintance with either English or American authors,—a deficiency which we are happy to find has been ably supplied by the editor and translator. By the profession in this country, it will be deemed sufficient evidence of the value of the work, that it has been considered by Dr. Meigs to be deserving of the time and attention which it has cost him to lay it before them. The task, to one whose time is so fully occupied, of translating such a work, and of adding extensive notes and comments, can hardly indeed be appreciated.

Dr. Meigs has added largely to his author. For notwithstanding the high estimate of the work expressed in his dedication and implied in the labour of translating it, he has not regarded the treatise of M. Colombat as sufficiently full on all points, or always correct. Sometimes he has added to the text, sometimes corrected its errors, and sometimes "protested" against its doctrines. In taking this liberty with his author, he acted properly—it was the course which the profession had a right to expect from one of his great experience. In saying thus much, we do not mean to be understood as taking sides on the subjects at issue between the author and his annotator. If we were to go at length into an examination of the opinions, pathological and therapeutical, advanced by them, we should perhaps be found sometimes on one side, and sometimes on the other, and occasionally differing from both. For instance, we should not agree with our friend and colleague in rejecting the tampon as a means of keeping hæmorrhage in check in cases of placenta prævia, nor in an almost exclusive reliance upon blood-letting, in the treatment of puerperal fever. These, however, with many others, are points of practice on which the ablest men of the profession are divided, and we have neither time nor space to discuss them at present.

In conclusion—we can very conscientiously recommend the present publication as containing a vast amount of information on the class of diseases of which it professes to treat. The translation, too, is of a different character from some we have had occasion to notice—M. Colombat actually appears in an English dress.

